# Role of Notch2 pathway in mature B cell malignancies

**DOI:** 10.3389/fonc.2022.1073672

**Published:** 2023-01-04

**Authors:** Nicolò Mesini, Stefania Fiorcari, Claudio Giacinto Atene, Rossana Maffei, Leonardo Potenza, Mario Luppi, Roberto Marasca

**Affiliations:** ^1^ Department of Medical and Surgical Sciences, Section of Hematology, University of Modena and Reggio Emilia, Modena, Italy; ^2^ Hematology Unit, Department of Oncology and Hematology, Azienda-Ospedaliero Universitaria (AOU) of Modena, Modena, Italy

**Keywords:** Notch pathway, Notch2, chronic lymphocytic leukemia, marginal zone lymphomas, drug-resistance

## Abstract

In recent decades, the Notch pathway has been characterized as a key regulatory signaling of cell-fate decisions evolutionarily conserved in many organisms and different tissues during lifespan. At the same time, many studies suggest a link between alterations of this signaling and tumor genesis or progression. In lymphopoiesis, the Notch pathway plays a fundamental role in the correct differentiation of T and B cells, but its deregulated activity leads to leukemic onset and evolution. Notch and its ligands Delta/Jagged exhibit a pivotal role in the crosstalk between leukemic cells and their environment. This review is focused in particular on Notch2 receptor activity. Members of Notch2 pathway have been reported to be mutated in Chronic Lymphocytic Leukemia (CLL), Splenic Marginal Zone Lymphoma (SMZL) and Nodal Marginal Zone Lymphoma (NMZL). CLL is a B cell malignancy in which leukemic clones establish supportive crosstalk with non-malignant cells of the tumor microenvironment to grow, survive, and resist even the new generation of drugs. SMZL and NMZL are indolent B cell neoplasms distinguished by a distinct pattern of dissemination. In SMZL leukemic cells affect mainly the spleen, bone marrow, and peripheral blood, while NMZL has a leading nodal distribution. Since Notch2 is involved in the commitment of leukemic cells to the marginal zone as a major regulator of B cell physiological differentiation, it is predominantly affected by the molecular lesions found in both SMZL and NMZL. In light of these findings, a better understanding of the Notch receptor family pathogenic role, in particular Notch2, is desirable because it is still incomplete, not only in the physiological development of B lymphocytes but also in leukemia progression and resistance. Several therapeutic strategies capable of interfering with Notch signaling, such as monoclonal antibodies, enzyme or complex inhibitors, are being analyzed. To avoid the unwanted multiple “on target” toxicity encountered during the systemic inhibition of Notch signaling, the study of an appropriate pharmaceutical formulation is a pressing need. This is why, to date, there are still no Notch-targeted therapies approved. An accurate analysis of the Notch pathway could be useful to drive the discovery of new therapeutic targets and the development of more effective therapies.

## Introduction

Notch signaling is an evolutionarily conserved pathway that modulates different cell-fate steps in a large number of multicellular eukaryotes. During the development of a single organism, the expression of Notch is properly regulated at precise times in a wide variety of tissues. The Notch signaling components have been characterized since pathway discovery, which happened at the beginning of the twentieth century. The Notch pathway is composed of a small number of molecules, in apparent contrast with its fundamental role, not just in fetal development but in continuous adult cell differentiation and self-renewal too. Although receptors and ligands are similar, they perform both common and specific non-redundant functions. Given that Notch plays a significant role in several cell types, illnesses affecting different organs and tissues are caused by Notch gene abnormalities, such as deletions and translocations. In fact, Notch signaling was also found active in tumor contexts where this pathway seems to have both oncogenic and oncosuppressor roles. The ambiguous nature of signaling is given by the fact that in some tissues it stimulates cell proliferation while in others it induces cell cycle arrest. Accordingly, neoplastic cells gain selective advantage through mutations, leading to a positive or negative modulation of the pathway. In particular, in mature B cell malignancies, Notch signaling has an oncogenic role because its activity seems to be related to upregulation of anti-apoptotic stimuli. However, future studies are necessary to fully understand Notch action in cell biology and its possible therapeutic alterations by innovative drugs.

## Notch characterization and signaling pathway

### The structure of Notch receptors

**Figure 1:**
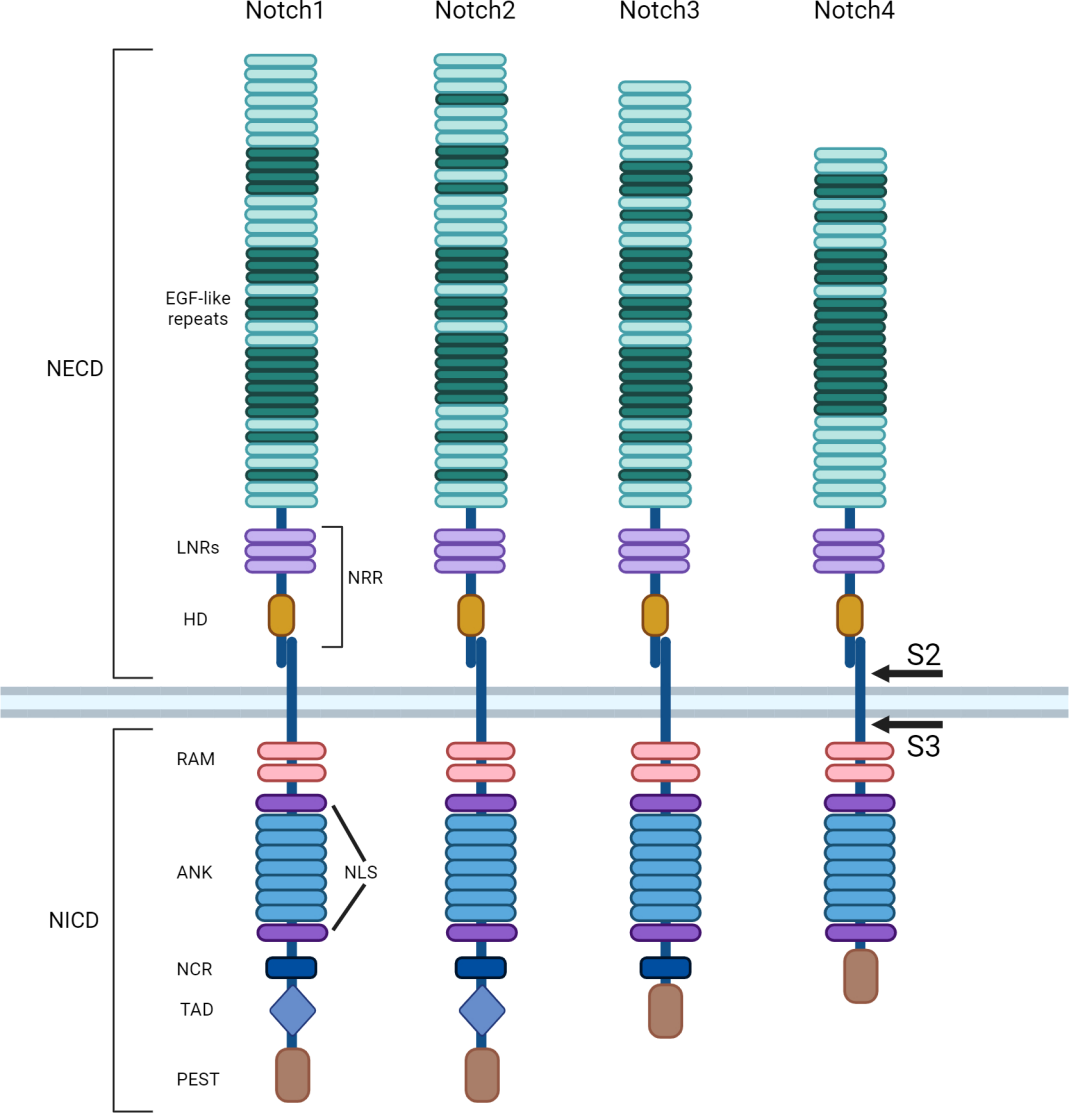
Notch receptors family. Notch receptors are single-pass type I transmembrane proteins. Mammalian cells have four Notch receptors (Notch 1-4). Mature receptors are heterodimers comprised of an N-terminal extracellular (EC) region, non-covalently linked to a transmembrane (TM) domain and a C-terminal intracellular (IC) subunit. The N-terminal portion presents several epidermal growth factor (EGF)-like repeats, followed by a juxtamembrane negative regulatory region (NRR), which contains three Lin12/Notch repeats (LNRs) and a heterodimerization domain (HD). After the TM, the common IC portion of the receptors consists of a protein-binding RBPJ-associated molecule (RAM) and two nuclear localization sequences (NLS) interposed by seven ankyrin repeats (ANK). The Notch cytokine response region (NCR) is absent in Notch4 receptor. The transcriptional activation domain (TAD) is present only in Notch1 and 2 receptors. The last portion of all receptors is the C-terminal PEST domain, rich in proline, glutamate, serine and threonine. After ligand binding, Notch is cleaved first by metalloproteases in the cleavage site S2, then it is rapidly further cleaved by the γ-secretase complex in S3.

Mammals have four Notch receptors (Notch1–Notch4) with both common and unique functions. They are hetero-oligomers with a large N-terminal extracellular portion (EC) linked in calcium-dependent and non-covalent contact with a transmembrane (TM) domain and a small C-terminal intracellular (IC) region (**Figure 1**) (1). These single-pass type I transmembrane proteins are synthesized as single precursors, undergo post-translational modification in the endoplasmic reticulum (ER), and mature in the Golgi apparatus after being cleaved by a furin-like proprotein convertase. Despite significant structural homology, Notch receptors show some differences. While Notch3 and Notch4 contain respectively 34 and 29 epidermal growth factor (EGF)-like repeats, Notch1 and Notch2 both have 36 repeats. Some EGF-like repeats are crucial to correctly mediating ligand interactions and responses. A portion of the EC domain is also critical to preventing Notch receptor activation in the absence of ligands (2, 3). This area consists of the juxtamembrane negative regulatory region (NRR), with its three Lin12/Notch repeats (LNRs) and the heterodimerization domain (HD). Other differences between Notch receptors concern the IC domain composition. The Notch cytokine response region (NCR) is absent in Notch4 and is present only in Notch1 and 2. Two nuclear localization sequences (NLS), seven ankyrin repeats (ANK) and a protein-binding RBPJ-associated molecule (RAM) are commonly present in the IC domain of all Notch receptors. Instead, the transcriptional activation domain (TAD) is only found in Notch1 and 2. The last portion of the receptors is the C-terminal PEST domain, so called because it is rich in residues of proline, glutamate, serine, and threonine. It contains the substrate site recognized by E3 ubiquitin ligases, regulates receptor stability and degradation, and is required to terminate signaling (4).

### Notch ligands and signaling directions

Pathway activation occurs canonically upon interactions with two main Notch-ligand families: Delta-like and Jagged. Two Jagged ligands (JAG1 and JAG2) and three Delta-like ligands (DLL1, DLL3, and DLL4) are expressed in mammalian cells (5), and all are transmembrane proteins with EGF-like repeats. As a result, several receptor-ligand combinations are conceivable that may result in a variety of responses. Notably, the most structurally divergent ligand, DLL3, is incapable of activating Notch receptors in trans (6). In most cellular contexts, signal-sending cells and signal-receiving cells expose both the receptor and the ligand. This is due to the fact that ligands often trans-activate receptors on contacting cells and cis-inhibit receptors expressed on the same cell, resulting in an overall definite direction of Notch signaling. Although this is not always the case, cis-inhibition has been found to cause a downregulation of the Notch receptor at the cell surface (7). DLL3, which cannot perform its trans-activation function, can only operate as a cis-inhibiting ligand. Additionally, it has been demonstrated that the IC domains of Notch ligand and receptor exhibit competitive interactions (8, 9).

### Notch pathway activation

**Figure 2:**
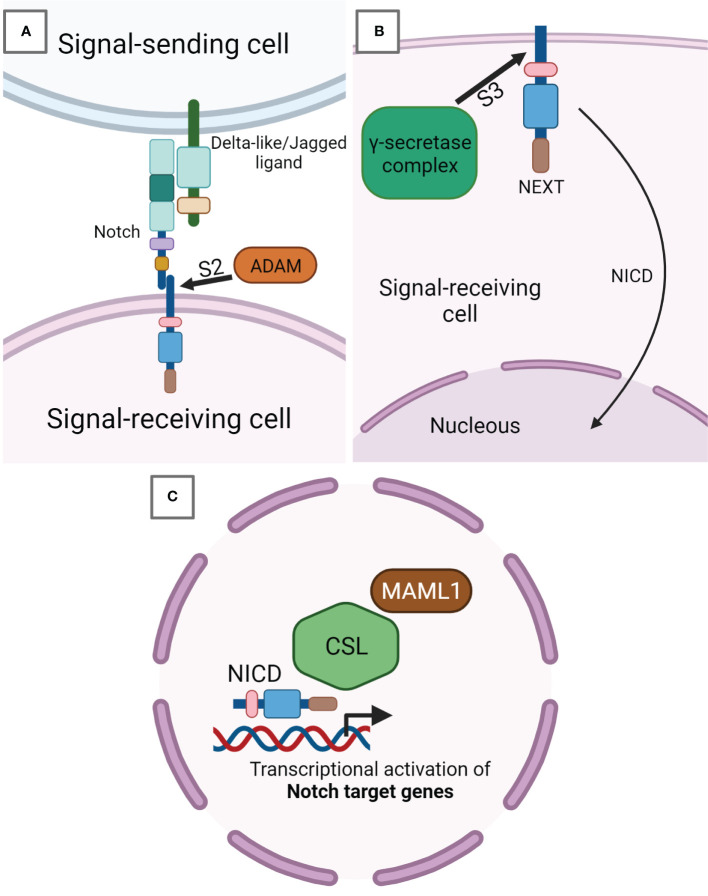
Notch signaling pathway. **(A)** Notch receptor expressed on signal-receiving cell surface binds to Notch-ligand Delta-like or Jagged exposed on the surface of the signal-sending cell. This binding induces a receptor conformational change, which leads to the cleavage in S2 by ADAM metalloproteases. ADAM-mediated cleavage releases the EC portion from the TM-IC subunit of Notch called NEXT (Notch extracellular truncated) and still anchored to the signal-receiving cell membrane. **(B)** Inside the signal-receiving cell, NEXT undergoes a further cleavage in the IC site S3 by the γ-secretase complex, after which the Notch IC domain (NICD) is released from the membrane and able of being translocated into the nucleus. **(C)** NICD in the nucleus activates the transcription of Notch target genes by inducing the development of a transcriptional complex having CSL as core DNA-binding factor. In this way, the CSL complex shifts its composition from a repressor to a transcription activator. In particular, NICD recruits the coactivator protein mastermind-like 1 (MAML1), which has a pivotal role in the start of Notch target genes transcription.

The binding of a Notch ligand on the Notch receptor expressed in neighboring cells induces a receptor conformational change which removes the inhibition applied by the NRR, exposing cleavage site S2 close to the TM domain to ADAM metalloproteases (**Figure 2A**). The signal-sending cell internalizes through endocytosis the first product corresponding to the ligand-Notch EC domain, facilitating the release of the second product constituted by the TM-IC domain also known as NEXT (Notch extracellular truncated) (10). The latter remains anchored to the signal-receiving cell membrane until the γ-secretase complex performs a further Notch cleavage at IC site S3, mediating Notch IC domain (NICD) release. Subsequently, NICD translocates into the nucleus thanks to its NLSs (**Figure 2B**) (11, 12). ADAM metalloprotease and γ-secretase complexes are able to further regulate the signaling of Notch cleaving also its ligands (9). Once in the nucleus, in particular, the RAM portion of NICD recruits a transcriptional complex in which RBPJ (also known as CSL in humans) represents the main effector of the DNA binding (**Figure 2C**) (13). NICD binding to CSL-containing complexes modifies their composition, shifting from a repressor to an activator of transcription. In particular, NICD mediates this conversion recruiting the coactivator protein mastermind-like 1 (MAML1), which is central to starting Notch target gene transcription. There are evidence that Notch ANK repeats are fundamental in MAML1 engagement. The Notch target genes are different depending on cell type and generally they belong to pivotal phases of cell biology, including cellular differentiation, metabolism, and cell cycle modulation. The basic helix-loop-helix (bHLH) transcription factor class represents the main Notch target genes. HES1 and HEY1 genes, for example, produce transcription repressors that act not only in fundamental stages of development but also in cancer onset (14). Under a direct Notch-dependent transcriptional control, there are also important transcription factors like Myc, which act as crucial transducers of Notch’s harmful effects in the neoplastic development of various cancers (15, 16). To arrest Notch signaling activity, the PEST domain is phosphorylated and subsequently ubiquitinylated by cyclin dependent kinase 8 (CDK8) and the E3 ligase complex containing FBW7, respectively. FBW7 is an F-box protein, crucial for NICD degradation, addressing Notch to the proteasome (17, 18). Besides, the Notch pathway establishes a negative feedback loop modulating transcriptionally cytoplasmic protein genes such as Deltex1 (DTX1) and NRARP, which inhibit NICD nuclear translocation or Notch binding to CSL (19, 20).

### Notch signaling features

The Notch signaling pathway plays a key role in virtually all species at many stages of development and in different tissues. This appears in contrast to the linear molecular architecture composed of few signaling components. In addition, it must be underlined that a single NICD molecule is generated from each activated and cleaved Notch receptor during its signaling. A stoichiometric relationship between signal input and output occurs because of a complete absence of an amplification step in signal transduction. Therefore, a proper cellular response depends on the balance of signal molecules (7). For these reasons, the Notch pathway is particularly susceptible to gene dosage variances. In fact, Notch2 or JAG1 human haploinsufficiency is observed in Alagille syndrome (21), a rare disease with a widely heterogeneous phenotype associated with vertebral malformations and heart defects (22, 23). A similar example is Notch1 haploinsufficiency seen in aortic valve disease (24). Another unique feature required for functional Notch signaling is the efficient ligand endocytosis by the signal-sending cell. The Notch ligand endocytosis is mainly regulated by the E3 ligase Mindbomb 1 (MIB1) (25, 26).

### Post-translational modifications of Notch receptors

**Figure 3:**
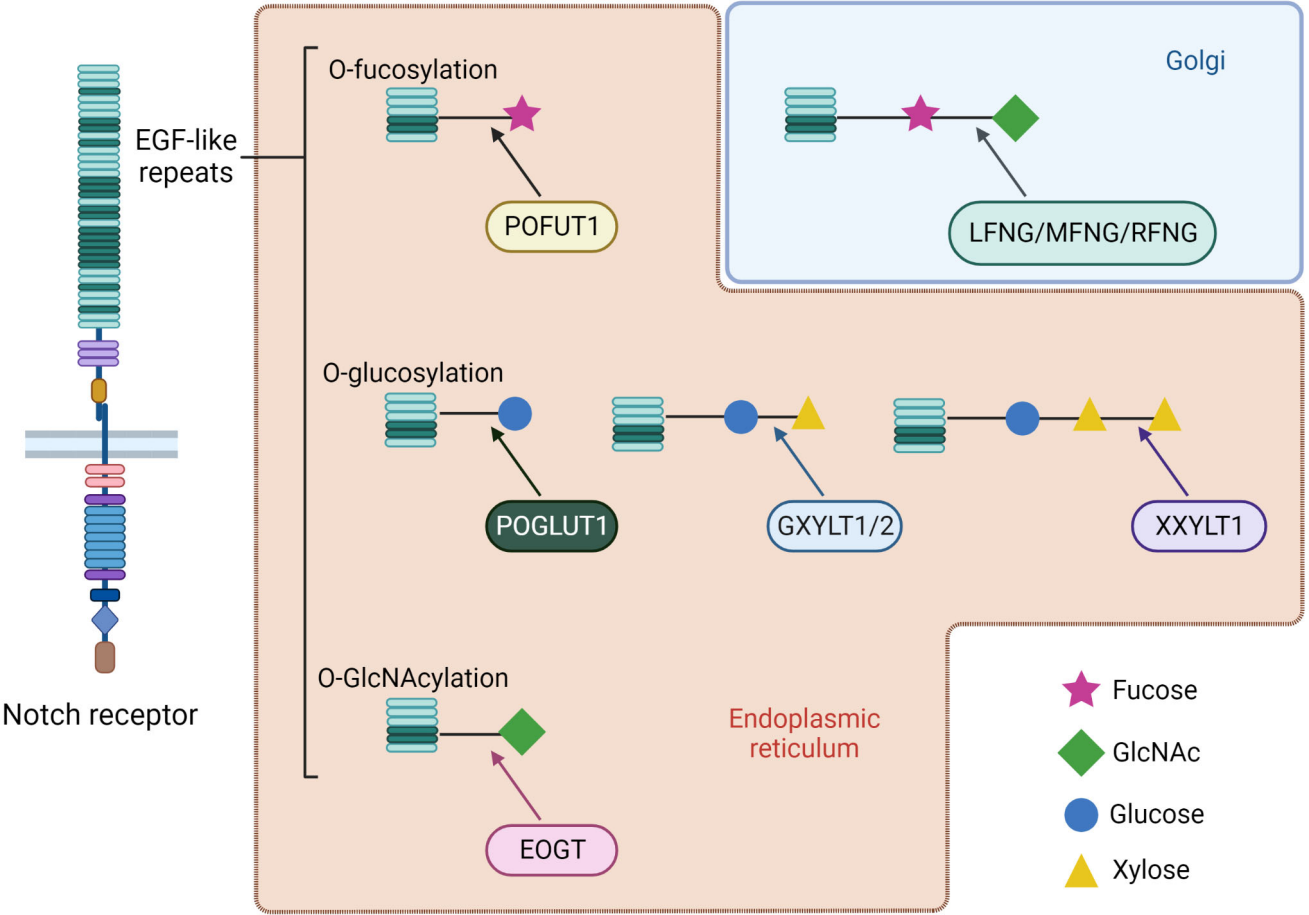
Types of receptors O-glycosylation and further post-translational modification of specific EGF-like repeats of Notch receptors. Most of the changes occur by ER enzymes during receptor maturation. An exception is represented by elongation of O-linked fucose with the addition of N-acetylglucosamine by the Fringe family glycosyltransferases, which are localized in the Golgi apparatus. Mammals have three homologs of *Drosophila* Fringe: Lunatic, Manic and Radical Fringe (LFNG, MFNG and RFNG). Depending on the physiological context, a modification can enhance receptor interaction with a specific family of ligands. At the same time, enzymes responsible for these modifications often have chaperon activity on Notch, hence representing fundamental proteins for correct trafficking and exposure.

The affinity between receptor and ligand and their consequent effects can be altered by Notch receptor post-translational modifications occurring in the ER or in the Golgi apparatus. Notch receptors can be modified by adding O-fucose, O-glucose or O-GlcNAc (N-Acetylglucosamine) residues to a serine or threonine within different consensus sequences of specific EGF-like repeats localized in the EC region, which can in turn lead to further modifications (**Figure 3**). I) The O-linked fucose modification is mediated by the ER protein O-Fucosyltransferase 1 (POFUT1). The Lunatic, Manic, and Radical Fringe (LFNG, MFNG, and RFNG) enzymes are mammalian homologs of the *Drosophila* Fringe protein (27). These are glycosyltransferase localized in Golgi that elongate O-linked fucose modification by adding a β1-3N-acetylglucosamine. In adult tissues, POFUT1 is ubiquitously expressed and its deletion results in severe embryonic defects in mice. In addition, POFUT1 plays a key role in Notch trafficking, acting as a chaperone protein necessary to translocate the receptor from the ER and enable its exposition on the cell surface (28, 29). Binding assays performed on Notch1 demonstrate that LFNG, MFNG, and RFNG make it more prone to being activated by DLL1 by enhancing DLL1-Notch1 binding. Contrariwise, LFNG and MFNG inhibit Notch1 signaling activated by JAG1, while RFNG enhances JAG1-mediated activation. Anyway, all three Fringe proteins enhance JAG1-Notch1 binding, suggesting that JAG1-Notch1 signaling inhibition by LFNG and MFNG is not attributable to reduced binding, but to some other downstream event still unknown (30, 31). II) Protein O-Glucosyltransferase 1 (POGLUT1) is the mammalian homolog of *Drosophila* Rumi enzyme, which is able to add an O-linked glucose residue to Notch receptors (32). This modification can also be further extended by the addition of a first xylose residue thanks to the *Drosophila* enzyme Shams, which has two mammalian homologous glucoside α3-xylosyltransferases (GXYLT1 and GXYLT2). Then a second xylose residue can be linked by the xyloside α3-xylosyltransferase (XXYLT1) (33, 34). Enzymes responsible for Notch O-glucosilation and successive xylosylations are all localized in ER (35). Also, POGLUT1 mutant mice die at an embryonic stage, reporting even more severe defects than those seen in Notch mutated mice (36). This suggests that POGLUT1 activity is necessary for correct development, but other targets of this enzyme are still unknown. III) Lastly, Notch could also be O-GlcNAcylated thanks to a specific extracellular O-GlcNAc Transferase (EOGT). EOGT mutants do not manifest notable phenotypes attributable to Notch in flies, hence the purpose of this Notch receptor modification remains unclear (37, 38). In humans, EOGT mutations have recently been related to a rare disease called Adams-Oliver syndrome, usually associated with aplasia cutis congenital and terminal limb defects (39, 40).

## History and physiological roles of Notch

Notch was discovered in the early 1900s thanks to two *Drosophila* geneticists, Dexter and Morgan, who were studying the alleles responsible for specific phenotypes on flies wings (41, 42). Flies harboring Notch receptor mutations had indentations on their wings, hence the name “notches”. Similar wing phenotypes were subsequently associated with mutations affecting Notch pathway genes like Delta or Jagged.

**Figure 4:**
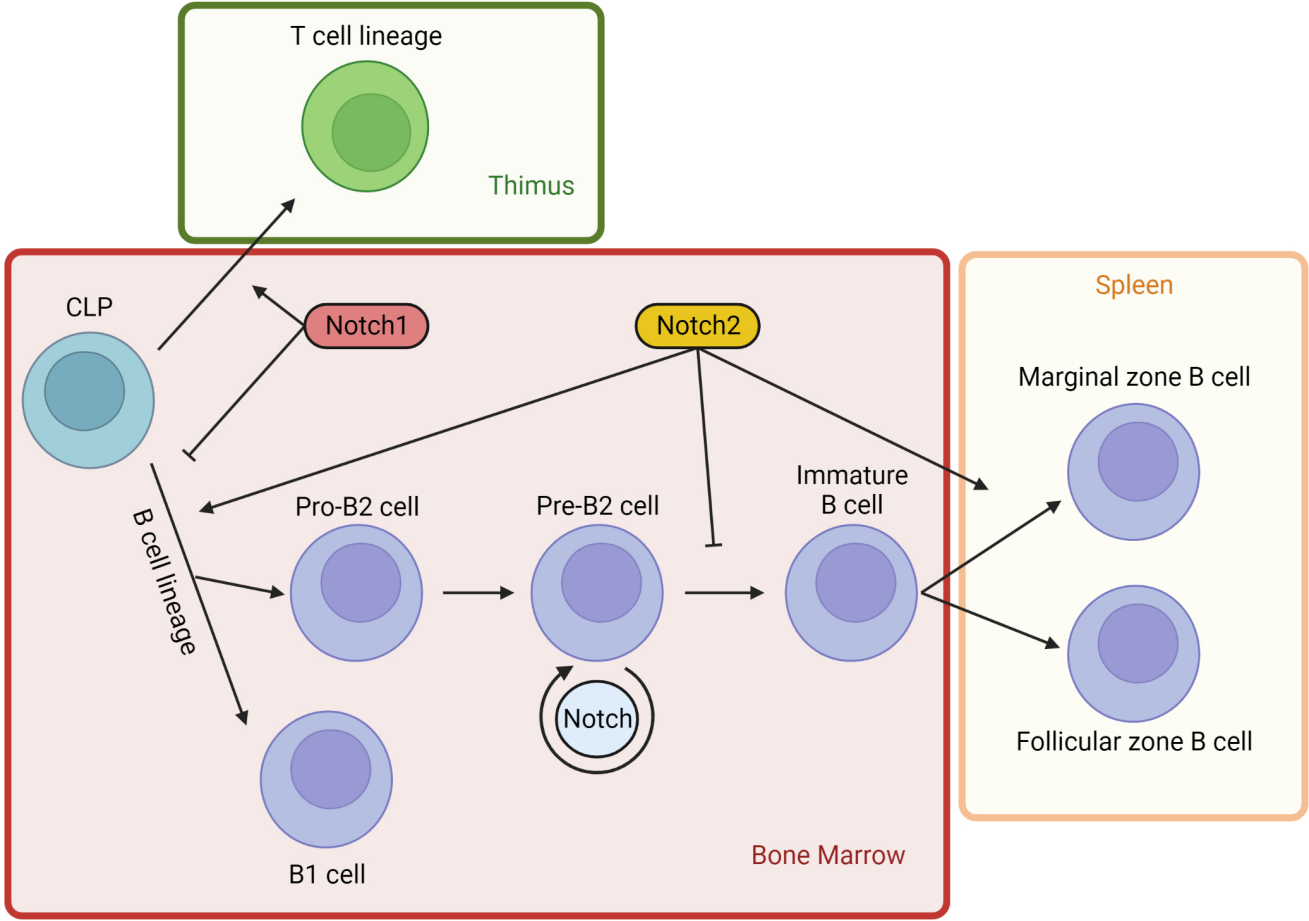
Notch signaling in lymphopoiesis. Notch activity influences multiple lineage decisions of developing lymphoid cells. Lymphopoiesis starts in the BM from CPLs (common lymphoid progenitors). A part of BM progenitors moves through the bloodstream into the thymus, where Notch1 activity stimulates T cells development from CLPs. Contrariwise, lineage B commitment is inhibited in CLPs by Notch1 pathway. Early B cell development from CLPs is induced by Notch2 signaling, independently of B1 or B2 branching. However, Notch2 activity blocks B2 cell progression at pre-B stage. Developing B cells can themselves express DLL, establishing a dynamic balance that maintains some lymphocytes at a precursor state. DLL1 is the most important physiological Notch2 ligand and it is not expressed by BM cells. DLL1 presence at high concentrations in splenic venules induces immature B cells homing to the spleen, where Notch2 activation DLL1-mediated induces immature B2 cells to differentiate into marginal zone B cells.

Thereafter, it was demonstrated that Notch signaling pathway components are fundamental and basically highly conserved in virtually all multicellular organisms (43). In humans, Notch activation has been detected in various lineages from embryonic stem cells to adult cells during development (44, 45). In particular, Notch influences multiple lineage decisions of developing lymphoid cells (**Figure 4**). Focusing on B lymphopoiesis, it has been characterized that B cells in the bone marrow (BM) differentiate from common lymphoid progenitors (CLPs), then they continue toward pro-B cells or branch into the B1 subset. Simultaneously, progenitors move through the bloodstream from the BM to the thymus, where they are stimulated to differentiate into a T cell lineage (αβ or γδ T cells), prior to relocating to the periphery. T cell commitment of CLPs is mediated by Notch1-DLL4 interaction at the expense of B cell development (46). Indeed, mice with inducible inactivation of Notch1 result in T cell differentiation blockade together with ectopic B cell accumulation in the thymus. Similar variations in mouse phenotype are obtained by manipulating the expression of transgenic dominant-negative forms of MAML-1 or Notch modulators such as Fringe family proteins and DTX1 (47, 48). DLL4 is the main Notch ligand expressed on thymic epithelial cells (TECs), and it seems to have an essential nonredundant role during T cell lineage commitment. In fact, specific DLL4 inactivation in TECs resulted also in the complete absence of T cell differentiation, coinciding with ectopic B cell thymic accumulation (49). Contrary to T cell development, commitment of CLPs into the B lineage needs the inhibition of Notch pathway. Indeed, B cell progenitors grow in a BM niche where stromal cells produce CXCL12, a cytokine that inhibits the surrounding expression of Notch ligands. Shortly after, the activity of the Notch pathway becomes necessary for the subsequent development step of progenitors B. The B cells migrate in secondary lymphoid organs towards a microenvironment expressing Delta-like and Jagged ligands and devoid of CXCL12 (50, 51). In addition, developing B cells can themselves preferentially express Delta-like ligands, in contrast to BM stromal cells, which express mainly Jagged ligands. It is possible that in BM, the expression of Delta-like ligands by early B cells at analogous developmental stages establishes a dynamic balance in which some lymphocytes are stimulated to rest in a precursor state while others are directed toward independent differentiation (52).

### Notch2 specific function in B cells development

Notch2 regulates two major checkpoints in the B cell lineage: B1 cells and marginal zone (MZ) B2 cell development. The B cell fate boundaries branch after the CLP state, restricting the following development of B progenitors toward a specific B cell lineage. In fact, CLPs are able to develop into B1 cells but other B progenitors, such as pro-B cells, are not (53). Indeed, in mice harboring Notch2 haploinsufficiency, there is a severe reduction of B1 cells and a complete absence of B2 cells (54). Instead, BM cells transduced with activated NICD2 to induce specific Notch2 signaling show a pronounced and selective increased development of B1 cells with the corresponding block of B2 lineage at the pre-B stage (55).

Early-stage lymphocytes committed to the B2 lineage, called pro-B cells, start the rearrangements of their immunoglobulin heavy-chain genes in BM. The pre-B cell receptor checkpoint assesses whether pro-B cells are successfully rearranged. After some proliferative cycles, these cells differentiate into immature B cells, rearranging their immunoglobulin light-chain genes. Immature B cells can further differentiate in the spleen into either follicular or MZ B cells. Follicular B cells are recirculating lymphocytes that participate in T cell-mediated immune responses. They generate germinal center B cells which, after class switching and somatic hypermutation, produce high-affinity antibodies and memory B cells. Most of the spleen is composed of follicular B cells, whose name originates from the follicles in which they reside. By contrast, MZ B cells are localized between the marginal sinus and the splenic red pulp at the border of the white pulp surface. Their role is to rapidly drive effective T cell-independent responses against blood-borne pathogens producing low-affinity IgM antibodies against encapsulated bacterial and polysaccharide antigens (56, 57). The initial homing to the spleen of many immature B cells might be linked to DLL1 expression. In fact, DLL1 is the main physiological Notch2 ligand responsible for its activation, and it is widely expressed in splenic venules but not in the BM (58). The induction of Notch2 signaling in mature follicular cells is sufficient to convert them into MZ B cells. The downregulation of transcription factor KLF2, which delimits the MZ division, represents the key point of this trans-differentiation downstream of Notch2 signaling (57). In light of this, Notch2 plays a pivotal role in the dynamic balance between follicular and MZ B cells.

MZ B cells are absent in mice with CD19+ B cells that are Notch2-deleted (54), suggesting that DLL1-Notch2 interaction also induces immature B2 cells to differentiate into MZ B cells. On the other hand, the true cell type through which DLL1-Notch2 signaling triggers MZB development is still unclear. In addition, for MZ B cell development, MIB1 activity is also required, because it regulates DLL1 endocytosis on the signal-sending cell surface (59). Likewise, knockout mouse B cells lacking RBPJ, MAML1 or DLL1 have defects in MZ B cell development. On the contrary, mice harboring mutations in genes connected to the inhibition of the Notch pathway lead to amplified MZ B cell development at the expense of follicular cell number. For example, the protein MINT competed with the RAM domain of the NICD, suppressing the transactivation activity of Notch signaling, so mice with inactivation of MINT have an increased number of MZ B cells (54, 60–64).

## Aberrant Notch signaling in hematological cancer

Beyond Notch physiological action, the pathway has been characterized as being deregulated in tumors, in particular in hematological malignancies. The first study reporting the presence of Notch mutations in tumors was about Notch1 translocations identified in the early 1990s in patients with acute lymphoblastic T cell leukemia (T ALL) (65). More than half of T ALL patients harbor Notch1 mutations affecting two NICD1 regions fundamental to its activation and transcriptional effects. The impairment of the NRR region exposes the receptor S2 cleavage site to ADAM metalloprotease action without the requirement of ligand binding. Moreover, PEST domain mutations inhibit NICD1 degradation, prolonging its transcriptional activity (66). In a similar way, another subgroup of T ALL patients harbors mutations in the regulatory FBW7 gene, resulting once again in prolonged NICD1 half-life (18). After that, other Notch pathway irregularities acting in hematopoietic and solid tumors were discovered. To date, it has been characterized as both tumor-suppressive and oncogenic roles of Notch. Unregulated signaling activation or inhibition, receptor or ligand overexpression, epigenetic silencing, and defective posttranslational modifications such as receptor and ligand fucosylation (5, 67, 68), and ubiquitination are all examples of pathway mutations (17, 69).

The tumor microenvironment (TME) is fundamental to cancer cell survival, establishing a bidirectional dialogue between neoplastic and normal cells. Tumor cells receive anti-apoptotic stimuli and develop drug resistance, while surrounding cells can be manipulated to induce angiogenesis and immunoescape in the TME. For example, Notch controls spouting angiogenesis physiologically in endothelial cells though DLL4 binding. Notch ligand JAG1 has an opposite function on endothelial cells, so its overexpression in neoplastic contexts promotes tumor vascularization (70, 71). Interestingly, extracellular vesicles (EVs) of multiple myeloma cells interact with surrounding BM cells, carrying mainly Notch2 receptors and a lower number of Notch1. Notch2-EVs have negative prognostic significance because they are associated with disease progression due to osteoclastogenesis and angiogenesis (72). A careful characterization of Notch pathway is useful because it also concerns tumor-associated macrophages (TAMs) development. CSL deletion in monocytes blocks their TAM differentiation and consequent immunosuppressive function, but at the same time, forced Notch1 activation in macrophages inhibits TAM activity, stopping tumor growth (73, 74). The use of γ-secretase inhibitors (GSI) reduces tumor angiogenesis and TAM and Treg populations (75, 76), similarly to anti-JAG1-2 antibodies treatment (77). Nevertheless, other studies show a Treg enhancement due to Notch signaling inhibition (78, 79), providing further evidence of the ambivalent role of the pathway.

Focusing exclusively on mature B cell malignancies, Notch1 is definitely the first and best characterized actor, but recent studies analyzing Notch2 activity have shown its relevant role.

## Notch2 in chronic lymphocytic leukemia

Chronic lymphocytic leukemia (CLL) represents the most common leukemia in western countries, with an incidence of 4–5 per 100,000 populations. CLL is diagnosed in 72-year-old patients on average, with almost double male dominance than women (80, 81). An increased risk of acquiring CLL and other lymphoproliferative disorders exists in the relatives of CLL patients, suggesting a still unknown hereditable genetic susceptibility (82, 83). CLL disease can be widely heterogeneous, but its fundamental feature is the clonal proliferation of mature, typically CD5+/CD19+/CD23+ B cells accumulating within the BM, blood, spleen, and lymph nodes. Although lymphocytes in the peripheral blood are predominantly resting, a small fraction of them are actively proliferating in specific structures localized in the lymph nodes and in the BM known as proliferation centers (84, 85).

Notch1 is one of the most commonly mutated genes detected at diagnosis in CLL patients (12%), and this frequency gradually increases in chemorefractory and advanced-stage patients (86, 87). In most cases, Notch1 lesions are deletions in exon 34, which generate a reading frame shift and a premature stop codon, leading to PEST domain truncation. As seen in T ALL, an irregular PEST domain prevents NICD1 degradation by the E3 ubiquitin ligase FBW7, causing an abnormally stabilized Notch signaling. The 3% of Notch1 non-mutated patients harbor FBW7 inactivating mutations, leading to similar effects on this pathway (88–90). Clinically, Notch1-mutated patients correlate with shorter overall survival (OS) and an increased risk of Richter Syndrome transformation (91).

One of the initiating chromosomal aberrations in CLL is the chromosome 12 trisomy (+12). Trisomy 12 is identified in 10–20% of CLL patients (92) and its acquisition occurs in about 30% of those who develop Richter syndrome (93). Patients harboring trisomy 12 correlate with more aggressive disease, higher risk of leukemia progression, considerable lymph nodal accumulation, higher Notch1 mutation incidence (94, 95), and low IRF4 expression. In particular, during CLL development, IRF4 has a crucial negative regulatory role in Notch signaling (96), thus lower IRF4 expression correlates with increased Notch2 level. As a consequence, Notch2 overexpression has been correlated with durable transcription of antiapoptotic stimuli such as Mcl-1. In fact, the +12 CLL cells are characterized by a reduced response to pro-apoptotic treatments in relation to abnormal expression of Mcl-1 mediated by Notch2. As a consequence, oncogene Mcl-1 overexpression correlates with poor prognosis and both apoptosis and chemo-resistance in CLL patients (97). At the same time, rare CLL cases which harbors IRF4 activating mutations lead to direct upregulation of oncogene Myc conferring to leukemic cells a proliferative advantage (98).

The transmembrane glycoprotein CD23 is another common antigen exposed on CLL neoplastic cells surface. Physiologically, CD23 is transiently expressed on B lymphocytes as a marker of activation (99, 100). Microenvironmental molecules like PMA or IFN-γ can activate the protein kinase C (PKC), which strongly induces the transcription of the CD23 gene (FCER2) in CLL cells. In addition, CLL lymphocytes stimulated *in vitro* with PMA show increased resistance to apoptosis (101). Notch2 signaling in leukemic cells is implicated in FCER2 regulation and antiapoptotic Bcl-2 gene transcription (102). Notch2 signaling can be activated by PKC, hence in a ligand-independent manner, which explains resistance to GSI treatment. For this reason, PKC inhibition by RNA interference or rottlerin (PKC selective inhibitor) downregulates Notch2 signaling and, consequently, CD23 expression in CLL cells, inducing apoptosis (101). The TME is necessary to leukemic cell survival, providing anti-apoptotic and immunosuppressive stimuli. The cross-activation of CLL cells and BM-derived mesenchymal stromal cells (BMSCs) leads to Notch2 signaling activation in stromal cells, which is necessary for subsequent Wnt signaling activation in leukemic B cells. Myc and cyclin D1 are target oncogenes of Wnt/β-catenin, hence in CLL also this pathway could be important for cell proliferation and disease progression. Anyway, it is necessary to have a better understanding of this mechanism because about a quarter of all primary CLL samples in co-culture express β-catenin independently of Notch2 pathway activation. At the same time, other Notch receptors have been shown to be unable to activate Wnt signaling in CLL-activated BMSCs, suggesting a nonredundant role of Notch2 in this cross-talk (103).

Myc is also a Notch target, through which cells of the BM and lymph node microenvironment induce the glycolytic shift in CLL cells (104). In addition, Myc directly promotes the expression of PD-L1, enhancing the immunoescape of leukemic cells, in particular in stromal niches (105). PD-L1 inhibits the function of innate and adaptive immunity cells, and its expression is stimulated also by IFN-γ. By secreting IFN-γ, CLL cells create an anti-apoptotic autocrine loop, and Notch upregulates both the cytokine and its receptor (106). Notch1-2 signaling represses genes fundamental to antigen-processing and presentation in CLL and mantel cell lymphoma cells, reducing their immunogenicity and having a negative impact on the prognosis of patients (107). The bivalent role of this pathway is re-emphasized by the fact that Notch2-DLL1 signaling is necessary in cytolytic CD8 T cell activation through perforin and granzyme B expression (108).

The Notch ligands JAG1 and JAG2 are constitutively expressed on CLL cells, suggesting the presence of both autocrine and paracrine signaling which constitutively activates the Notch pathway, promoting leukemic cell survival and chemo-resistance. In fact, JAG1 stimulation is associated with an increase in NF-kB activity, which subsequently leads to the upregulation of antiapoptotic genes such as c-IAP2 and XIAP (cellular and X-linked inhibitor of apoptosis protein, respectively). As a consequence, the down-regulation of these antiapoptotic proteins through Notch inhibition correlates to enhanced apoptosis. Notch seems to act as a direct regulator of c-IAP1 and c-IAP2 expression as well as a protein stabilizer, interacting directly with XIAP (109). In addition, when the number of NICDs in the nucleus increases, normal epigenetic regulation is impaired. In particular, other genomic regions, including the CD20 promoter, can be silenced by free HDAC accumulation mediated by intensified Notch target gene transcription. Accordingly, alterations of Notch signaling reduced patients benefits from anti-CD20-based chemoimmunotherapy regimens compared to wild type samples (110).

## Notch2 in splenic and nodal marginal zone lymphoma

MZ B cell development is critically modulated by the Notch2 activity. Mutations affecting Notch2 pathway are recurrently seen in marginal zone lymphomas (MZLs) (111, 112). Between lymphoma categories, MZLs are a subgroup arising from memory B cells in secondary lymphoid follicles and specifically in the “marginal zone”. In adults, they represent about 5-17% of all non-Hodgkin lymphomas. In addition, MZLs are further categorized into three different subtypes in dependence of the main disease localization: extranodal, splenic, or nodal. Considering all MZL cases, splenic MZL (SMZL) represents about 20% of them, while nodal MZL (NMZL) is the least common, amounting to only 10%. In recent decades, clinical, etiological, and pathological heterogeneity among these kinds of lymphomas have been studied (113). SMZL is a malignancy arising from mature B cells and which concerns the spleen, BM, and peripheral blood. In particular, within the spleen, neoplastic cells are localized around the MZ germinal centers, but these small lymphocytes also infiltrate the red pulp. Instead, NMZL is a rare, indolent, and nodal-localized B cell neoplasm with a different pattern of dissemination than SMZL, without clinical evidence of extranodal or splenic involvement (114).

Notch2 mutations occur in both SMZL and NMZL in about 20% of cases. Similarly to CLL, frequent Notch2 mutations affect the NICD2 PEST domain, enhancing protein half-life and deregulating pathway signaling. A lower portion of non-extranodal MZL harbors mutations involving negative regulators of Notch signaling, like SPEN and DTX1, or members of the Notch2 transcriptional complex, for example, MAML2. Interestingly, Notch pathway mutations seem to be mutually exclusive between different tumor samples. It is possible to suppose that each mutation has been alternatively selected during neoplastic cell evolution and exploited in a similar fashion (111, 115, 116). Physiologically, in B cells, SPEN inhibits Notch activity, acting as a negative regulator of MZ B cell development (64). In particular, the SPEN C-terminal domain is fundamental for interacting with RBPJ and inhibiting its activity as a transcription coactivator together with NICD, but the majority of SPEN mutations truncate specifically this critical region. In a similar way, MZ B cells highly express DTX1, which seems to regulate important changes during B lymphocyte differentiation. The DTX1 gene encodes a RING finger ubiquitin ligase which modulates Notch signaling activity by binding receptors belonging to this family (47). Mutations affecting DTX1 are found especially in SMZL and they involve regions of the RING finger necessary for its correct interaction and functioning (111). Despite this, in MZLs, downstream targets of Notch signaling are still unclear. For example, recent studies have demonstrated that Notch2 is able to regulate other fundamental cellular pathways such as PI3K/AKT and NF-kB. Notch2 oncogenic effects could be mediated by these downstream pathways, but further analyzes are necessary to evaluate this possible cross-talk (117). On the other hand, the therapeutic inhibition of Bruton tyrosine kinase (BTK), a crucial component of B cell receptor signaling, is not influenced by Notch2 mutational status (118). To date, Notch2 mutations in MZLs have been associated with a higher risk of disease progression (119).

## Notch pathway therapeutic targeting

The discovery of oncogenic role for Notch signaling in numerous cancers has led to the development of various approaches targeting distinct pathway steps. Nevertheless, there are no Notch-targeted therapies approved yet. Since Notch signaling starts with receptor proteolytic cleavage to generate free NICDs, therapeutic strategies are being evaluated to block this cleavage. In particular, the γ-secretase complex is the crucial point for Notch signaling cascade. In fact, GSIs efficiently block the activity of the pathway. Moreover, monoclonal antibodies capable of recognizing Notch receptors and preventing ligand binding or subsequent exposure of cleavage sites are very effective. Nevertheless, systemic Notch signaling inhibition leads in “on-target” gastrointestinal toxicity in animal studies. This is due to the imbalance of the intestinal differentiation stimuli that increases the amount of secretory goblet cell to the detriment of proliferative cell (120, 121). Specific inhibition of only one type of receptor reduces or avoids this effect, hence the use of selective antibody targeted therapy is clearly better than pan-Notch inhibitors (122). GSI-induced gastrointestinal toxicity can be attenuated by the combinatorial use of glucocorticoids *in vivo* because they induce cyclin D2 expression, which stimulates proliferative cell development and partially re-establishes gastrointestinal differentiation balance (123).

The monoclonal antibody OMP-52M51 has been isolated from mice immunized with human Notch1 fragments including the LNR and the HD receptor domain. *In vitro* and *in vivo*, it efficiently inhibits Notch signaling and reduces Notch activation T ALL samples harboring Notch1 or FBW7 mutations (124). Tarextumab is a fully human IgG2 monoclonal antibody that acts as a Notch2 and Notch3 antagonist with minimal binding to Notch1. It successfully inhibits the interaction of Notch2 with its ligands, DLL4 and JAG1 (125). However, Tarextumab has shown inconsistent survival benefits in pancreatic cancer patients, but there have been no clinical trials on hematological patients (126). On the other hand, antibody targeting specifically Notch2 receptor-NRR nearly eliminated CD21^high^ splenic MZ B cells, interfering with ADAM metalloprotease cleavage in S2. By contrast, antibodies against NRR1 do not result in the same effect on the MZ population, showing that it is Notch2 who plays the pivotal role in this lineage point.

In recent years, it has also been evaluated as a therapeutic strategy a fungal secondary metabolite, gliotoxin. Gliotoxin strongly reduces splenic B cell viability and in CLL cells inhibits Notch2 transactivation, inducing apoptosis. Its molecular mechanism is still unknown, but it seems to be NF-kB-independent and it may be mediated by Notch3 up-regulation (127). Synthetic α-helical peptides that mimic the protein MAML1 (SAHM1) are another experimental Notch signaling inhibition strategy. SAHM1 has no MAML1 active domain able to recruit the CSL complex, so it inhibits the transcriptional effect of Notch pathway binding to NICD. Human T ALL cell lines and mouse models treated with SAHM1 result in strong Notch-specific inhibition of cell proliferation and leukemia progression (128). Despite these findings, the use of synthetic α-helical peptides for therapeutic purposes remains limited.

To date, there is no clinical data available for specific Notch targeting in MZL patients (129).

In conclusion, the importance of the evolutionarily ancient Notch pathway is undeniable. Notch signaling has a pivotal role in a variety of physiological processes, but also in tumor onset and progression. For this reason, a better understanding of the Notch pathway is desirable and useful. The analysis of signaling members still not fully characterized could refine current therapies. Further studies in order to better define the Notch pathway and to propose more effective drugs or drug combinations are necessary. Future potential therapeutic strategies against Notch2-related B cell malignancies would target specifically its pathway in tumor cells, reducing side effects of treatments. In particular, it is important that the developing drugs preserv physiological role of Notch signaling in normal cells. The main aim of further research effort will be to provide new therapeutic models which improve life quality and extend Notch-induced cancer patients survival.

## Author contributions

NM reviewed the literature and wrote the manuscript. SF, CA and RosM collaborated to review the literature, contributed to the discussion and revised the manuscript. RobM, PL and ML supervised and critically revised the manuscript. All authors contributed to the article and approved the submitted version.
